# Supplementary material for: Fast electrical modulation of strong near-field interactions between erbium emitters and graphene
Source: Nat Commun. 2020 Aug 14;11:4094. doi: 10.1038/s41467-020-17899-7 (PMC7427803; doi:10.1038/s41467-020-17899-7)
Supplement: Supplementary file 1 — Supplementary Information [file 41467_2020_17899_MOESM1_ESM.pdf]

Supplementary Information

Fast electrical modulation of strong near-field interactions between erbium emitters and graphene

Cano et al.

Supplementary Note 1. Emission properties of erbium-doped thin films

Removing the intrinsic non-radiative decay from the erbium-doped Y_2O_3 films is an essential requirement for the accurate experimental evaluation of the energy transfer at the erbium-graphene interface. The non-radiative losses due to defects that are usually present in nanoscale rare-earth-doped crystals constitute a competing energy flow channel for the erbium-graphene interactions, leading to undesired emission quenching. We overcome this problem by using a fabrication procedure that we have optimized to produce few-nanometer-thick erbium-doped Y_2O_3 films with almost pure radiative decay. In this fabrication procedure, the erbium-doped Y_2O_3 films are grown by atomic layer deposition (ALD), see Scarafagio *et al.*[1] for details, with an optimized annealing post-treatment that eliminates the non-radiative decay channels. With this technique, we are able to exploit the many advantages of ALD, such as accurate thickness control at atomic scale, good uniformity and the ease to vary the erbium doping level, while preserving the high optical quality of the erbium emitters.

For this work, we first did preliminary tests with erbium-doped Y_2O_3 films on Si(100), for different thicknesses and annealing parameters, and determined that optimal annealing occurs at 950 °C for 2 hours. Using these parameters, we produced a series of ten thin film samples made of a 11-nm-thick Y_2O_3:Er (2%) film grown on a 285-nm-thick SiO_2 layer of a p-type silicon wafer. This oxide layer serves as electrical isolation between the graphene devices and the backgate. Afterwards, on six of the ten thin film samples we grew an additional Y_2O_3 capping layer with a thickness of either 1 nm or 2 nm, and we annealed the samples again at 600 °C for 2 hours. Supplementary Figure 1a shows the measured lifetimes of the ten thin film samples, where we can see the beneficial impact of the capping layer in reducing the non-radiative decay channels. In order to verify that the non-radiative processes due to surface defects are negligible, we compared the decay curves of the thin film samples with the decay curve of a bulk reference sample of erbium-doped Y_2O_3. Except in two thin film samples without capping layer, the decay curves were almost mono-exponential and had similar, or even longer, lifetimes as compared to the bulk reference. As a bulk reference we used a ceramic prepared by high-temperature solid-state reaction. For this, high purity Y_2O_3 and Er_2O_3 were mixed and annealed twice with intermediate crushing at 1500 °C for 24 hours.

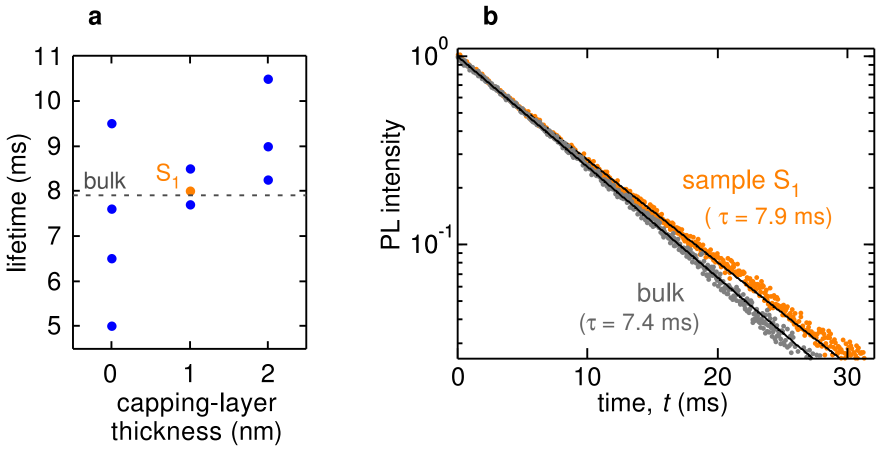

Supplementary Figure 1: Comparison between thin-film samples and the bulk reference sample. **(a)** Lifetimes of ten different thin film samples consisting of a 11-nm-thick Y_2O_3:Er (2%) film grown by ALD on a SiO_2/Si wafer, with subsequent annealing at 950 °C for 2 hours. Six thin film samples had an additional Y_2O_3 capping layer, annealed at 600 °C for 2 hours, the thickness of which was 1 nm in three samples and 2 nm in the other three. These measurements suggest that the capping layer reduces the probability of non-radiative decay channels. The dashed line shows the lifetime of the reference bulk sample. **(b)** Decay curves of the thin film sample S_1 (before depositing graphene) and of bulk erbium-doped Y_2O_3. The experimental data is described by a mono-exponential curve of lifetime τ (black solid line).

For the main study, we chose a thin film sample with similar emission properties as the bulk reference material. Supplementary Figure 1b shows the decay curve of the thin film sample, which we call S_1, together with the decay curve of the bulk reference material. The similarity between the two almost mono-exponential decays is an evidence of the negligible effect of the surface defects in sample S_1. The slightly longer lifetime in the thin film sample is the result of the lower effective refractive index of the thin film environment in comparison with bulk Y_2O_3. The significant improvements of the thermal post-treatment are shown in Supplementary Fig. 2 and Supplementary Table 1, which compare the decay-rate probabilities of sample S_1 with other thin film samples made without optimized thermal post-treatment. In S_1, the rate at which de emission decays to 1/e is $\gamma_{1/e} \sim 134$ Hz, just a bit larger than the decay rate of the electric and magnetic dipole moments, $\gamma_{ed} + \gamma_{md} \sim 125$ Hz (Supplementary Reference 2). This shows that the decay is dominated by radiative processes. Furthermore, the full width half maximum (FWHM) of the decay rate distribution is only 33 Hz, much lower than $\gamma_{1/e}$. This

is the evidence that the ion ensemble is highly homogeneous and that the exponential decay curves are hardly affected by detrimental effects such as non-radiative decay channels, erbium-erbium interactions and energy migration to quenching centers. For this reason, to simplify the simulations of the N-ion model described in Methods, we can make the approximation that the non-radiative decay rate is $\gamma_{\rm nr} \sim 10$ Hz for all ions of this sample.

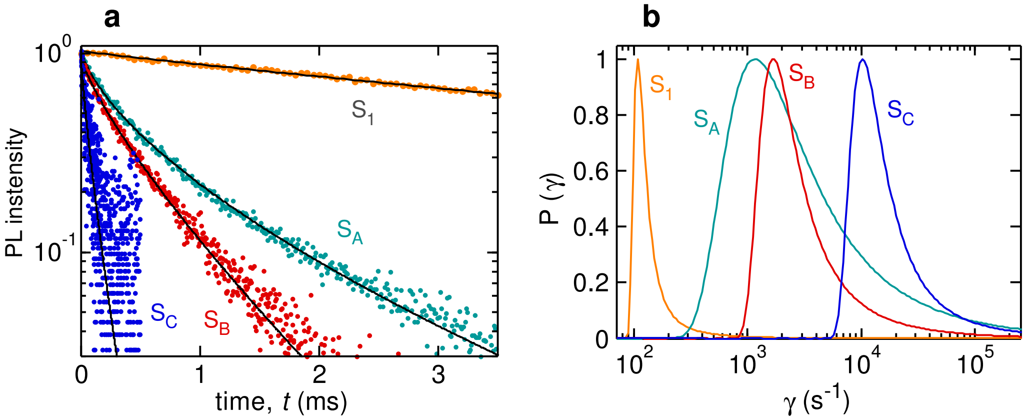

Supplementary Figure 2: Effect of optimization post-treatment on decay curves. Comparison between the thin film sample S_1 (used for the device of the main text) and three thin film samples made without optimal annealing post-treatment. **(a)** Measured decay curves. The black solid lines are the best-fit stretched exponential functions. **(b)** Decay-rate distributions, $P(\gamma)$, obtained by inverse Laplace transformation of the experimental decay curves (see Methods). The distributions are normalized to their respective maxima for a clearer comparison.

thin film sample	Y_2O_3 thickness (nm)	annealing temperature (°C)	optimization post-treatment	$\gamma_{1/e}$ (Hz)	FWHM (Hz)
S_1	11 (+1 nm capping)	950	Yes	134	33
S_A	40	800	No	2136	3977
S_B	11	800	No	2613	2020
S_C	11	No	No	15500	10700

Supplementary Table 1. Effect of optimization post-treatment on the decay rates. Fabrication parameters and fitting parameters of the four thin film samples, where $\gamma_{1/e}$ is the rate at which de emission decays to 1/e ~ 0.368, and FWHM is the full width half maximum of $P(\gamma)$. The sample S_1, in which FWHM is only 33 Hz, has an almost mono-exponential decay. Only S_1 has a capping layer.

Supplementary Note 2. Characterization of the erbium-graphene hybrid devices.

We used a scanning confocal microscope setup[3] to create emission maps of the devices (see Supplementary Fig. 3). This was done by scanning a CW excitation laser at 532 nm over the device and measuring the emitted light at 1545 nm. The area of the graphene monolayer is easily distinguished by the emission quenching effect caused by graphene. We confirmed the graphene monolayer with the Raman spectrum, shown in Supplementary Fig. 4a.

The emission contrast between the regions with and without graphene was used together with the measured decay curves to extract the distribution of decay-enhancement factors, $P(F_{\mathrm{P}})$, as described in Methods. The numerical procedure to extract $P(F_{\mathrm{P}})$ is valid only if the relationship between erbium emission and excitation laser power is linear. This occurs when the excitation laser power, P_{exc}, is sufficiently low to avoid saturation of the the erbium transitions. To verify this, we measured the photon emission as a function of P_{exc}, as shown in Supplementary Fig. 4b. We can see that for the laser power used in our experiments, $P_{\mathrm{exc}} = 0.2$ mW, the relationship between emission and P_{exc} is approximately linear, as required. The experimental data is described by the function $(P_{\mathrm{exc}}/P_{\mathrm{sat}})/\left[1 + (P_{\mathrm{exc}}/P_{\mathrm{sat}})\right]$ (see Supplementary Reference 4), thus obtaining the saturation laser power $P_{\mathrm{sat}} = 0.9$ mW (we ignore the dependence of P_{sat} on the excitation laser spot size because this is the same in all our experiments: $\lesssim 1\mu$m).

Having a low laser power is also important to rule out the possibility of collective erbium-graphene interactions. The erbium concentration is 2% ($\simeq 10^{21}$cm^{-3}), which means that approximately one hundred ions are contained in the plasmon mode volume ($\sim \lambda_{\mathrm{pl}}^3$). Using our N-ion model (see Methods) and the value of $P_{\mathrm{exc}}/P_{\mathrm{sat}}$ of our experiments, we have estimated that the average erbium population in the excited state is $\sim 0.2\%$ in the samples with graphene. This implies that there is less than one excited ion within the plasmon mode volume, thus the erbium-graphene interactions can be described by single-ion physics.

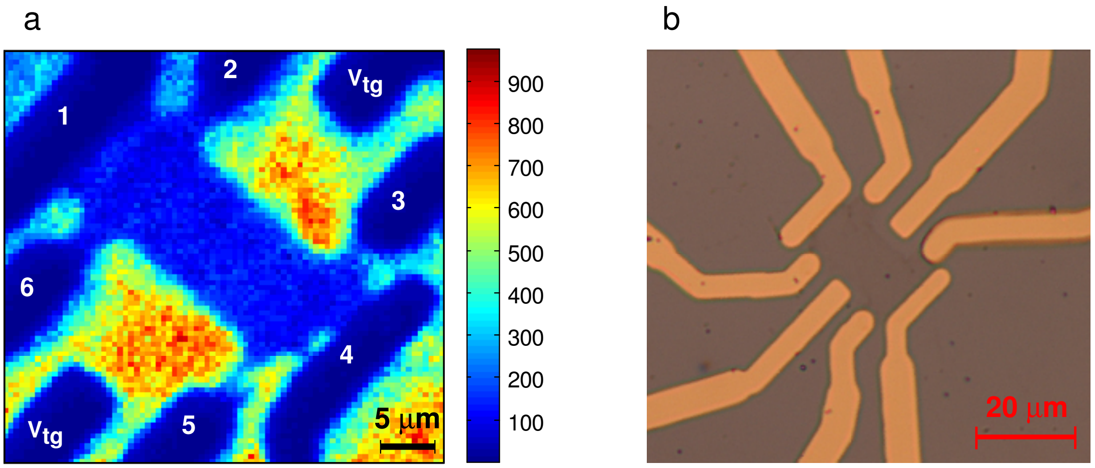

Supplementary Figure 3: Central area of the erbium-graphene device. **(a)** Emission map of the device used in the main text. The central part of the device is a monolayer of CVD-grown, wet-transferred graphene on the erbium-doped thin film S_1. Graphene is patterned into a Hall-bar geometry by means of photolithography. The device contains six Cr/Au electrical contacts to graphene (numbered 1 to 6) and two Cr/Au electrical contacts to apply the topgate voltage V_{tg} to the polymer electrolyte. The color scale indicates emission in counts per second. Clearly, emission is quenched by the presence of graphene, which is the result of near-field energy transfer from excited erbium ions to graphene. **(b)** Optical microcope image of a device.

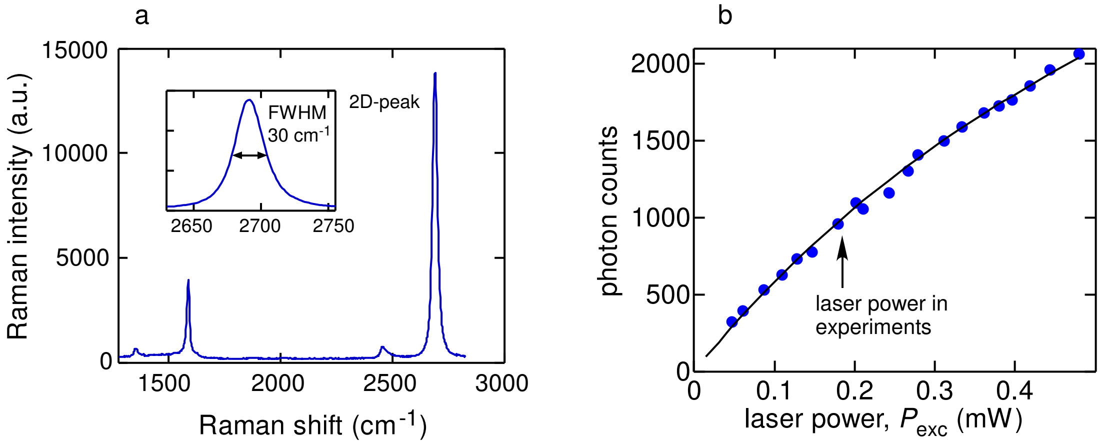

Supplementary Figure 4: Preliminary tests of the sample. **(a)** Graphene characterization using Raman spectroscopy. The FWHM of the 2D peak is approximately 30 cm^{-1}, which confirms the presence of the single layer graphene. **(b)** Photon counts as a function of excitation laser power in the sample S_1 without graphene. The relationship between emission and P_{exc} is approximately linear around 0.2 mW, as required for the correct interpretation of emission measurements.

Supplementary Note 3. Relationship between the Fermi energy and the applied gate voltages

It is well established that the two different near-field emitter-graphene coupling regimes occur at different Fermi energies in graphene: below ~ 0.4 eV, coupling occurs through interband transitions in graphene, whereas above ~ 0.6 eV, coupling occurs by intraband transitions, leading predominantly to plasmons. Between 0.4 eV and 0.6 eV, there is an intermediate regime in which both intraband and interband processes are reduced, which leads to the maximum of emission of far field photons (see Fig. 2a of the main text). In order to confirm that we can statically and dynamically modulate the near-field emitter-graphene coupling between the interband and intraband regimes, we verified the Fermi energy induced by the applied gate voltages for both top and back gates. For this task, we performed a series of independent measurements, including Hall measurements and device resistance measurements.

We performed Hall measurements in order to determine the relationship between the Fermi energy, E_{F}, and the topgate voltage, V_{tg}. These Hall measurements were carried out in a separate setup, where we applied a current of $I_{\mathrm{bias}} = 1$ μA between the contacts 1 and 4 and measured the Hall voltage between contacts 2 and 6 (see Supplementary Fig. 3), under application of a magnetic field normal to the device surface. We measured the Hall voltage vs. topgate voltage for a positive and a negative field of $B_{+/-} = \pm 0.85$ T, obtaining transverse voltages V_+ and V_-, respectively. We then obtained the graphene carrier density using $n = I_{\mathrm{bias}}(B_+ - B_-)/e(V_+ - V_-)$. By taking the difference for positive and negative perpendicular magnetic field, we removed possible spurious transverse voltages from sample asymmetry. The Fermi energy was obtained from $E_{\mathrm{F}} = \hbar v_{\mathrm{F}}\sqrt{\pi n}$, where v_{F} is the Fermi velocity. We found that the Fermi energy generated by the topgate can be approximated by $E_{\mathrm{F}} \sim E_{\mathrm{F},0} + AV_{\mathrm{tg}}$, with $A = 0.2$ eV V^{-1}. This linear relation is valid above $E_{\mathrm{F}} \sim 0.3$ eV (see Supplementary Fig. 5a). The Fermi energy at zero gate voltage $E_{\mathrm{F},0}$ varies significantly from device to device as it is determined by the amount of charge impurities (see Supplementary Fig. 5b). Therefore, the slope A is the most important parameter we extracted from the Hall measurements. In agreement with previous studies, we find that the Fermi energy induced by the polymer electrolyte gate cannot be approximated by a straightforward capacitive coupling constant (which would give a carrier density that is linear in gate voltage rather than a Fermi energy linear in gate voltage).

For the dynamic control of the erbium-graphene interactions, we need to know the Fermi energy oscillation amplitude, $\Delta E_{\rm F}$, that is induced by the AC voltage amplitude applied to the backgate, $\Delta V_{\rm bg}$. Just before every dynamic modulation experiment, we found the relationship between $\Delta E_{\rm F}$ and $\Delta V_{\rm bg}$ by means of a procedure based on device resistance measurements. To illustrate our procedure, we show in Supplementary Figs. 5c-d the device resistance measurements that we did for the dynamic modulation experiments of Fig. 3 of the main text. We first measured the device resistance as a function of $V_{\rm tg}$ while keeping the backgate voltage at 0 V, as shown in Supplementary Fig. 5c. Then, we converted $V_{\rm tg}$ to $E_{\rm F}$ by using the calibrations described in the previous paragraph, thus obtaining the device resistance versus $E_{\rm F}$. Next, we tuned $V_{\rm tg}$ to the base Fermi energy of the corresponding dynamic modulation experiment. This was $V_{\rm tg} = 0.2$ V ($E_{\rm F} = 0.45$ eV) in the measurements of Fig. 3 of the main text, and $V_{\rm tg} = 2.2$ V ($E_{\rm F} = 0.8$ eV) in the measurements of Fig. 4 of the main text. Then, while keeping $V_{\rm tg}$ fixed, we measured the device resistance versus $V_{\rm bg}$, and converted the device resistance into Fermi energy using the measurement of Supplementary Fig. 5c, thus finding $\Delta E_{\rm F}$ versus $\Delta V_{\rm bg}$.

By means of numerical fitting, we found $\Delta E_{\rm F} \sim B\Delta V_{\rm bg}$, with $B = 15$ eV mV^{-1} (13 eV mV^{-1}) for a topgate-generated Fermi energy of 0.45 eV (0.8 eV), which corresponds to a capacitance per unit area of $C = 1.6 \cdot 10^{-3}$ Fm^{-2} ($2.4 \cdot 10^{-3}$ Fm^{-2}). This capacitance has been calculated using the expression $B = (\pi C\hbar^2 v_{\rm F}^2)/(2eE_{\rm F})$, where $E_{\rm F}$ is the base Fermi energy induced by the topgate voltage, and e is the electron charge. This expression comes from the first-order term of the Taylor expansion of the Fermi energy as a function of gate voltage[5]. It is remarkable that the experimental backgate capacitance of our devices is much larger than that expected for the 285-nm-thick SiO_2 layer of our substrate: $C_{\rm SiO_2} = 1.2 \cdot 10^{-4}$ Fm^{-2}. This indicates that the electrolyte in contact with graphene has an enhancement effect on the backgate capacitance. A similar enhancement effect has been observed in previous works [6,7], and is likely related to the large effective epsilon induced by the presence of the ions of the polymer electrolyte directly above the graphene layer.

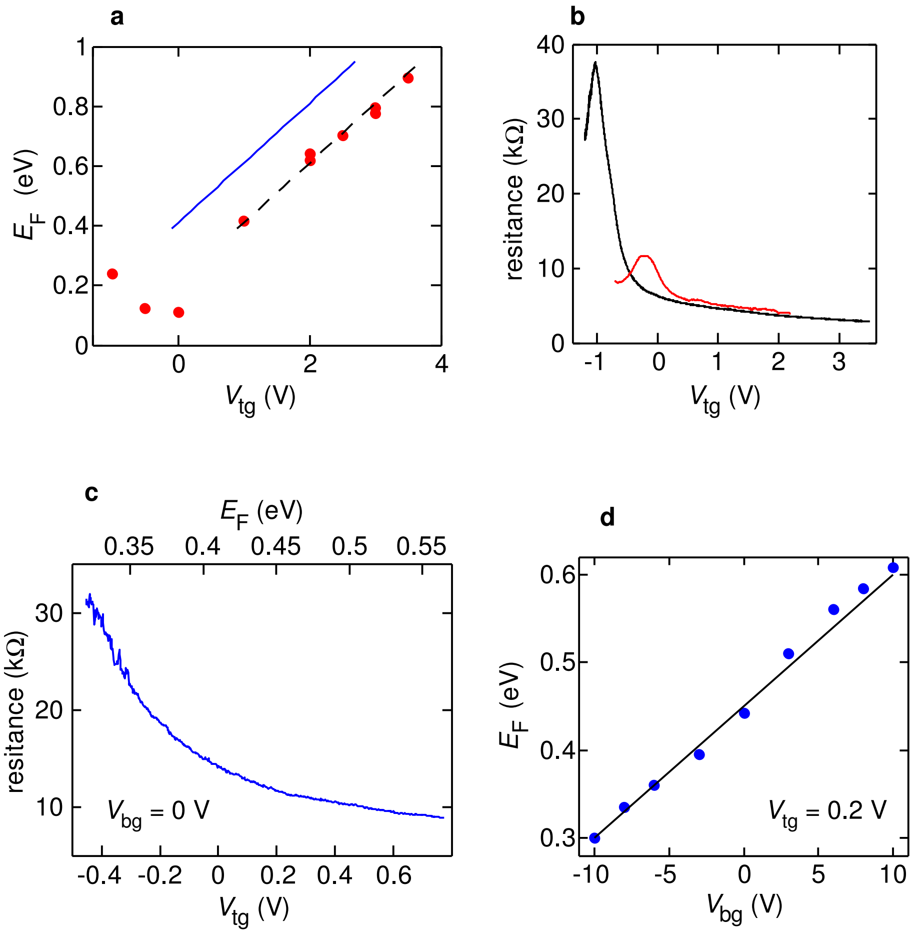

Supplementary Figure 5: Calibration of the Fermi energy of graphene. **(a)** Calibration of the Fermi energy as a function of the topgate voltage. The calibration is based on the Fermi energy versus topgate voltage obtained experimentally by means of Hall effect measurements (red dots). These measurements are described by a linear function, $E_{\rm F} \sim E_{\rm F,0} + AV_{\rm tg}$, where $A = 0.2$ eV V^{-1} (black dashed line). The Fermi energy at zero gate voltage $E_{\rm F,0}$ varies significantly from device to device. In the case of the device used in the main text, $E_{\rm F,0} = -0.41$ eV, and the corresponding linear function is plotted as a blue solid line. **(b)** Device resistance as a function of topgate voltage for two different devices, from which we can determine the Fermi Dirac point of the device. **(c)** Device resistance as a function of $E_{\rm F}$ just before modulation measurements of Figure 3 of the main text. The curve was obtained by measuring the device resistance as a function of $V_{\rm tg}$ for $V_{\rm bg} = 0$ V, and converting $V_{\rm tg}$ to $E_{\rm F}$ using the calibration from (a). **(d)** Fermi energy versus $V_{\rm bg}$, with fixed topgate, $V_{\rm tg} = 0.2$ V ($E_{\rm F} = 0.45$ eV). Here, the experimental points were obtained by measuring the device resistance for different backgate voltages, and converting device resistance into $E_{\rm F}$ using the curve of (c). The linear fit (black solid curve) has a slope of $B = 15$ mV eV^{-1}, which we use to know the Fermi energy in the experiments of Figure 3 of the main text.

We carried out dynamic modulation experiments for different backgate-voltage amplitudes, as shown in Supplementary Fig. 6, to confirm that we can modulate the Fermi energy between the interband and the intraband regimes. In these measurements, ΔV_{bg} was varied between 3 V and 10 V, while the topgate voltage was fixed at $V_{\mathrm{tg}} = 0.2$ V ($E_{\mathrm{F}} = 0.45$ eV). We theoretically simulated the emission by using our N-ion model (see Methods) with the ion density distribution of Fig. 2c of the main text. In each simulation, ΔE_{F} was a free parameter. Supplementary Figure 6e shows the best-fit values of ΔE_{F} for the different applied voltage amplitudes, ΔV_{bg}. The relationship between ΔE_{F} and ΔV_{bg} is approximately linear, with a slope of $B = 15$ eV mV^{-1}, the same value as obtained from the device resistance measurements described in the previous paragraph. This corroborates the calibration with the device resistance measurements.

We performed a dynamic modulation test to verify the transition from the interband regime to the intraband regime as the Fermi energy is increased. The test consisted in a series of measurements, shown in Supplementary Fig. 7, in which the base Fermi energy was increased from 0.4 eV to 0.7 eV using the topgate. A small modulation amplitude of $\Delta E_{\mathrm{F}} \sim \pm 60$ meV was applied using the backgate. We can see that, as the base Fermi energy increases, the oscillation inverts its sign, thus indicating the transition into the intraband regime, in which plasmon creation is the dominant decay mechanism of the erbium ions. This sign inversion corresponds to the change of the sign of the slope of the emission contrast shown in Fig. 2a of the main text.

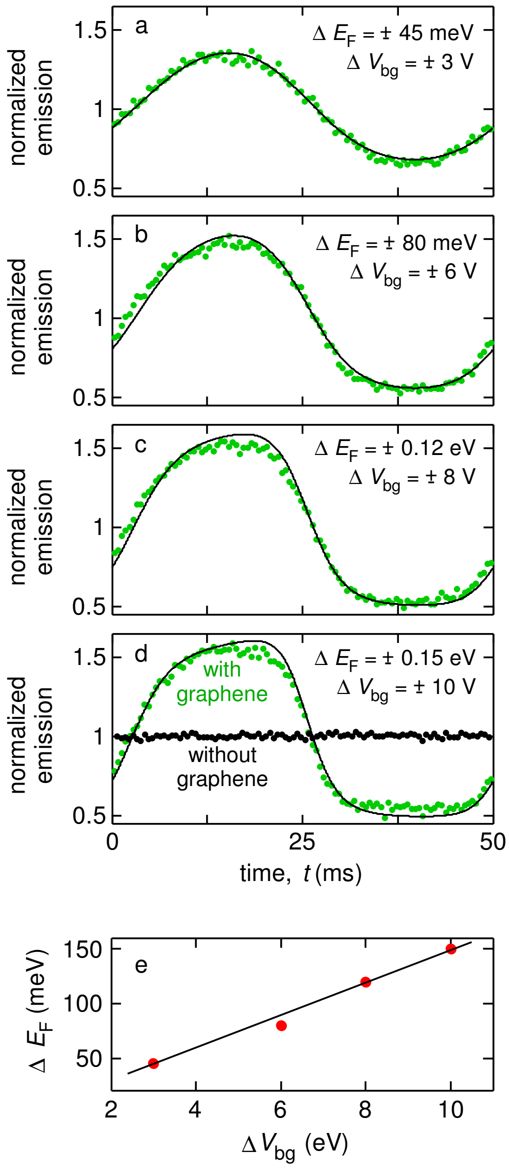

Supplementary Figure 6: Verification of the modulation between the interband and intraband regimes. **(a-d)** Dynamic modulation measurements (green dots) for different backgate modulation amplitudes, $\Delta V_{\rm bg}$, between ± 3 V and ± 10 V. In all measurements the topgate voltage is 0.2 eV, which corresponds to a base Fermi energy of $E_{\rm F} = 0.45$ eV. The solid black lines show the theoretical emission simulated using the N-ion model with $\Delta E_{\rm F} \sim BV_{\rm bg}$, where $B = 15$ eV mV^{-1}. **(d)** The emission measurement on graphene (green dots) is compared with the emission measurements when the excitation laser shines a region of the device without graphene (black dots). We use this comparison to confirm that emission oscillations are caused by graphene and not by any other effect induced by the gates. **(e)** Best-fit values of $\Delta E_{\rm F}$ obtained using the N-ion model with the oscillations of subfigures a-d.

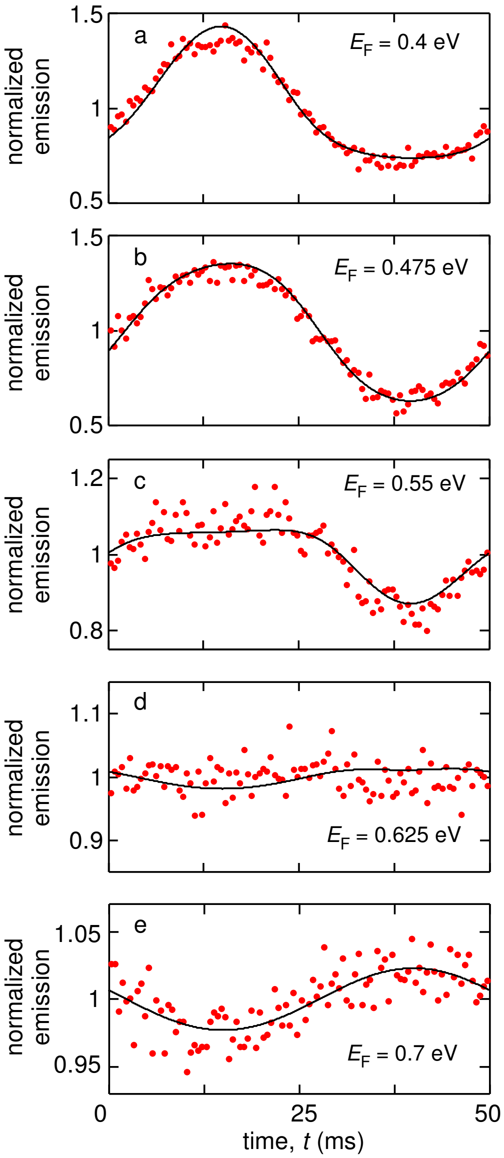

Supplementary Figure 7: Inversion of the modulation signal due to plasmons. Dynamic modulation measurements (red dots) for a Fermi energy modulation amplitude of $\Delta E_{\mathrm{F}} = \pm 60$ meV, induced by an AC backgate voltage at 20 Hz. The base Fermi energy induced by the topgate is **(a)** 0.4 eV, **(b)** 0.475 eV, **(c)** 0.55 eV, **(d)** 0.625 eV and **(e)** 0.7 eV. The sign of the oscillation inverts as the base Fermi energy increases, which is an obvious signature of the transition into the intraband regime in graphene. The black solid curves show the numerical simulation using the 50-ion model described in Methods.

We checked the Fermi energy during optical measurements by monitoring the device resistance (see Supplementary Fig. 8). We observed that the device resistance remains sufficiently stable for several hours, which indicates a high degree of stability of the Fermi energy induced by the applied gate voltages. This long-term stability allowed the realization of optical measurements during several hours without significant Fermi energy drifts.

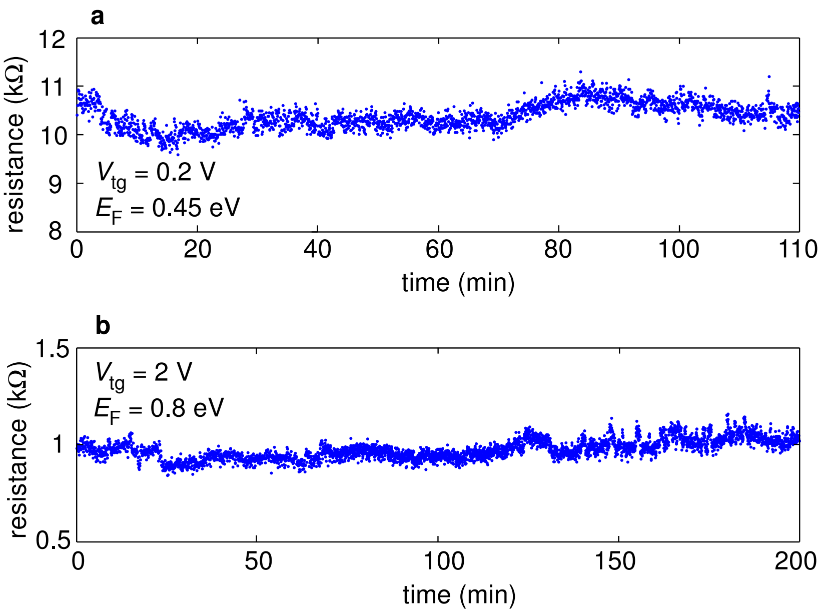

Supplementary Figure 8: Monitoring the stability of the Fermi energy. Measurements of the device resistance during optical measurements at Fermi energies of **(a)** 0.45 eV and **(b)** 0.8 eV. We applied a potential difference over the graphene sheet (typically, 2 mV) in order to monitor the device resistance and verify, in this way, that the Fermi energy remains stable during the measurements.

Supplementary Note 4. Results obtained with other erbium-graphene devices.

We obtained decay curves and emission maps from fifteen graphene devices on six different thin film samples. Furthermore, we performed dynamic modulation in three different devices on two different thin film samples (the first thin film samples, which were grown on Si(100), did not have a backgate). We measured similar erbium-graphene interactions in all devices. All of them showed a clear transition between the interband and intraband regimes. Supplementary Figure 9 shows the decay curves and the corresponding decay rate distributions of two devices.

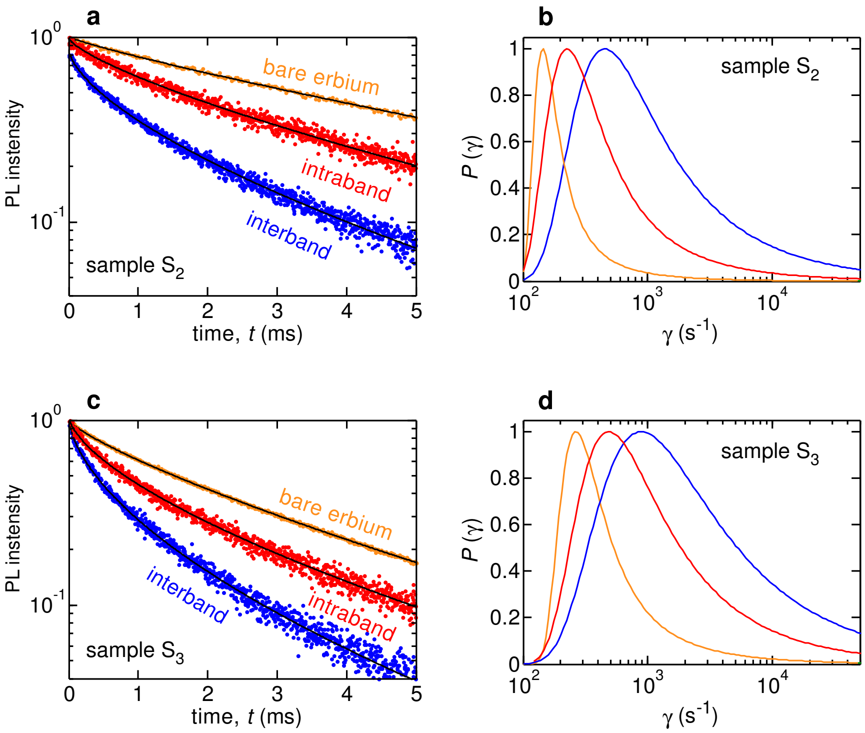

Supplementary Figure 9: Optical characterization of additional erbium-graphene devices. Measured decay curves and their corresponding decay rate distributions for two devices on two different thin film samples, that we denote as S_2 and S_3. **(a-b)** Results for the device on S_2, which was fabricated in the same way as S_1, but without the capping layer. **(c-d)** Results for the device on S_3, which was fabricated by growing a 11-nm-thick Y_2O_3:Er (2%) film on a Si(100) substrate and subsequent annealing at 1000 °C. For each device, we considered three cases: graphene in the interband regime ($E_F = 0.2$ eV, blue), graphene in the intraband regime ($E_F = 0.8$ eV, red), and without graphene (orange). The black solid lines correspond to the best-fit stretched-exponential curves. The decay rate distributions were obtained by inverse Laplace transformation of the measured decay curves (see Methods).

Supplementary Note 5. Maximum decay-enhancement factor that can be extracted from the decay curves.

The decay-enhancement-factor distributions, $P(F_P)$, and the density distribution, $P(z)$, were obtained by analyzing the experimental decay curves and the emission contrast measurements together, as described in Methods. The decay curves, $n(t)$, reflect mainly the dynamics of the ions with the lowest decay enhancements factors since these are the ions that emit the

largest amount of photons during lifetime measurements. Using the numerical techniques of Supplementary Reference 8, we determined the maximum decay-enhancement factor that can be extracted from the measured decay curves, $F_{\mathrm{P,max}} \sim 1,000$, which corresponds to an erbium-graphene separation of ~7 nm (see Supplementary Fig. 10a). To check that this value of $F_{\mathrm{P,max}}$ actually represents the accuracy limit provided by our experimental decay curves, we did a test which consisted in calculating the decay curves from $P(F_{\mathrm{P}})$ by doing the inverse numerical procedure that we did to calculate $P(F_{\mathrm{P}})$ from the experimental decay curves and emission contrast measurements (see Methods). In this test, we first converted $P(F_{\mathrm{P}})$ into $P(\gamma)$, and then we calculated the decay curves by numerically computing the Laplace transformation,

$$n(t) = \int_0^{\gamma_{\max}} \frac{P(\gamma)}{\gamma} \mathrm{e}^{-\gamma t} d\gamma, \tag{1}$$

where $\gamma_{\max}$ is the maximum decay rate considered in the decay curve. For this test, we neglected the intrinsic non radiative decay of the erbium-doped thin film. Supplementary Figure 10b shows the beginning of the decay curve of the device on the thin film S_1 in the interband regime, together with the decay curves calculated from $P(\gamma)$ for different $\gamma_{\max}$. As $\gamma_{\max}$ is increased, the calculated decay curves get closer to the experimental decay curves. We can see that the ions with $F_{\mathrm{P}} > 1,000$ have a very small effect which appears only in the first microseconds of the decay curves. To experimentally observe the contribution of these ions more clearly, we would need to reduce the histogram bin size as well as to increase the time of the measurements. However, given the very low photon emission of our devices, that would require weeks of continuous photon collection. It is more convenient to determine the distributions of the ions with $F_{\mathrm{P}} > 1,000$ by using the emission contrast measurements, as described in Methods. The maximum cutoff factor $F_{\mathrm{P,max}} \sim 1,000$ corresponds to an erbium-graphene separation between 5 nm and 8 nm, depending on the Fermi energy (see Supplementary Fig. 10a). As a trade-off, for our model we use 7 nm as the distance below which the density distribution is extracted from the emission contrast measurements. In this way, we obtain the erbium-graphene distances z_i (i = 1,...,50) shown in Supplementary Fig. 10c. The distances z_i are converted into the the density distribution $P(z)$ and vice versa by means of numerical integration and discretization, respectively.

It is interesting to remark the high F_P factors at short distances from graphene. As we can see in Supplementary Fig. 10a, F_{P} is higher than 10^6 at separations below 2 nm. This corresponds to energy transfer rates higher than $\gamma_{\mathrm{gr}} \simeq F_{\mathrm{P}}\gamma_{\mathrm{ed}} \sim$ 75 MHz, where γ_{ed} ~75 Hz

for erbium ions[2]. Nonetheless, these values do not represent the ultimate limit of the emitter-graphene interactions. In fact, $\gamma_{\rm gr}$ can be further increased by more than one order of magnitude by patterning the graphene monolayer into waveguides and cavities with defined plasmonic modes [9, 10, 11] or by using a graphene-insulator-metal heterostructure[12]. Such high values of $\gamma_{\rm gr}$ can be modulated with graphene, in which $f_{\rm mod}$ can be up to tens of GHz[13] (In general, $\gamma_{\rm gr}$ determines the maximum modulation frequency that the ion dynamics can follow). The experimental observation of such high modulation frequencies may involve thinner erbium thin films and a configuration based on graphene plasmon cavities with optical nanoantennas or waveguides[14] that enhance the far-field emission of the ions with very large $\gamma_{\rm gr}$.

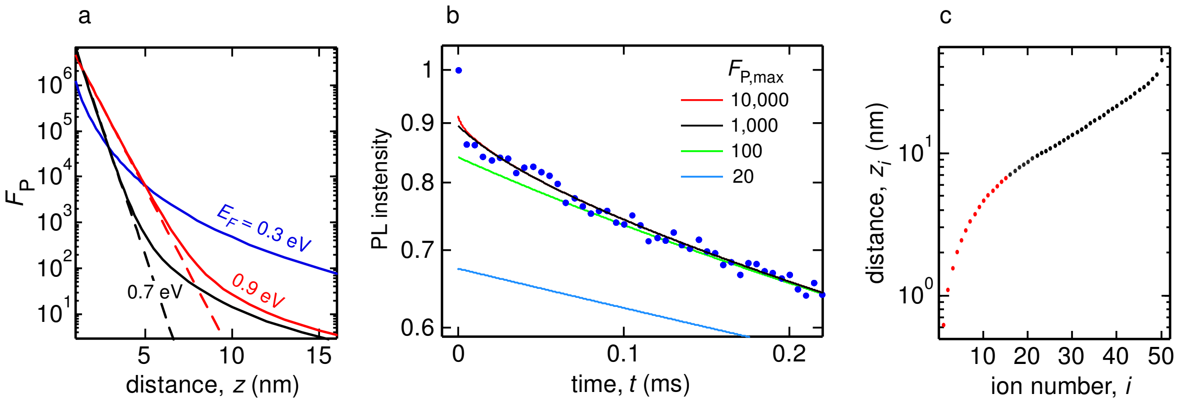

Supplementary Figure 10: Evaluation of the maximum decay-enhancement factor. (a) Decay-enhancement factor $F_{\rm P}(z)$ as a function of erbium-graphene distance for three different Fermi energies: $E_{\rm F}$ =0.3 eV (blue), 0.7 eV (black) and 0.9 eV (red). These theoretical functions have been calculated as in Supplementary Refs. 9 and 10, using the experimental parameters described in Methods. The dashed lines represent the ideal exponential decay, $F_{\rm P} \propto \exp(-4\pi z/\lambda_{\rm pl})$, of the long-distance propagating plasmons without losses, where $\lambda_{\rm pl} = 4.9$ nm (7.7 nm) for $E_{\rm F} = 0.7$ eV (0.9 eV). The deviations from the ideal plasmon exponential decay are caused by typical losses in CVD graphene. **(b)** Evaluation of the maximum decay-enhancement factor provided by the decay curves. The blue dots show the beginning of the measured decay curve of the device on thin film sample S_1 in the interband regime (see Fig. 2a of the main text). The solid lines are the theoretical decay curves calculated from $P(F_{\rm P})$ considering only the ions with $F_{\rm P}$ below a maximum cutoff factor, $F_{\rm P,max}$. **(c)** Erbium-graphene distances used to simulate the emission during dynamic modulation of the near field. The distances z_i are obtained either from the decay curves (black dots) or from the emission contrast (red dots).

Supplementary Note 6. Time-of-Flight Secondary Ion Mass Spectrometry (ToF-SIMS)

We obtained the density profile of the Er^{3+} ions by ToF-SIMS (see Methods). We determined the Er^{3+} density profile from the removed YO^- particles instead of from the ErO^- particles because the YO^- signal was much less noisy. The noise of the ErO^- signal was relatively high because we had to use the less abundant isotope ^{168}Er, with 27% abundance, in order to prevent overlap with other species. To verify that the YO^- signal provides a good estimation of the Er^{3+} density profile we plot in Supplementary Fig. 11 the density profiles obtained from both ErO^- and YO^- signals. In this figure we can see that the diffusion is practically the same for both species.

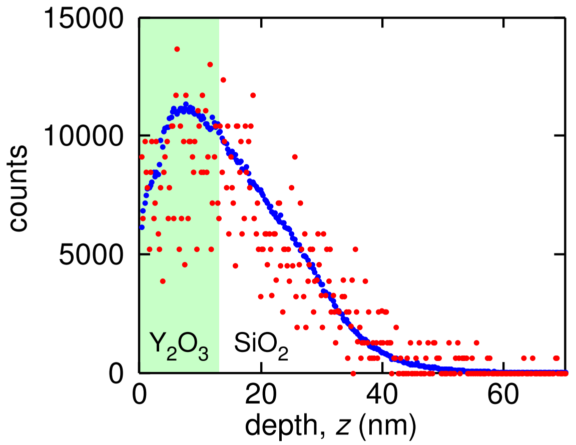

Supplementary Figure 11: TOF-SIMS measurements. Depth profiles of YO^- (blue dots) and ErO^- particles (red dots). The similarity between both profiles proves that the diffusion is very similar for both Y^{3+} and Er^{3+} ions. The Erbium signal has been multiplied by 650 for a clear comparison.

Supplementary References

1. Scarafagio, M. *et al.* Ultrathin Eu- and Er-Doped Y_2O_3 Films with Optimized Optical Properties for Quantum Technologies. *J. Phys. Chem. C* **123,** 21, 13354-13364 (2019).

2. Weber, M. J. Radiative and Multiphonon Relaxation of Rare-Earth Ions in Y_2O_3. *Phys. Rev.* **171,** 283 (1968).

3. Tielrooij, K., Orona, L., Ferrier, A. *et al.* Electrical control of optical emitter relaxation pathways enabled by graphene. *Nature Phys.* **11,** 281287 (2015).

4. Grynberg, G., Aspect, A., Fabre, C. Introduction to Quantum Optics: From the Semi-classical Approach to Quantized Light. *Cambridge: Cambridge University Press* (2010).

5. Castro Neto, A. H., Guinea, F., Peres, N. M. R., Novoselov, K. S. & Geim, A. K. The electronic properties of graphene, *Rev. Mod. Phys.* **81,** 109 (2009).

6. Xia, J. L., Chen, F., Wiktor, P., Ferry, D. K. & Tao, N. J. Effect of Top Dielectric Medium on Gate Capacitance of Graphene Field Effect Transistors: Implications in Mobility Measurements and Sensor Applications. *Nano Lett.* **10,** 50605064 (2010).

7. Grover, S., Joshi, A., Tulapurkar, A. & Deshmukh, M. M. Abrupt p-n junction using ionic gating at zero-bias in bilayer graphene. *Sci. Rep.* **7,** 3336 (2017).

8. Johnston, D. C. Stretched exponential relaxation arising from a continuous sum of exponential decays. *Phys. Rev. B* **74,** 184430 (2006).

9. Koppens, F. H. L., Chang, D. E. & Garca de Abajo, F. J. Graphene Plasmonics: A Platform for Strong LightMatter Interactions, *Nano Lett.* **11,** 8, 3370-3377 (2011).

10. Gonçalves, P. A. D. & Peres, N. M. R. An introduction to graphene plasmonics. Appendix P (World Scientific Publishing Co. Pte. Ltd., Singapore, 2016).

11. Christensen, J., Manjavacas, A., Thongrattanasiri, S., Koppens, F. H. L. & García de Abajo, F. J. Graphene Plasmon Waveguiding and Hybridization in Individual and Paired Nanoribbons. *ACS Nano* **6,** 431-440 (2012).

12. Alcaraz Iranzo, D. *et al.* Probing the ultimate plasmon confinement limits with a van der Waals heterostructure. *Science* **360,** 6386, 291-295 (2018).

13. Phare, C., Lee, Y.H.D., Cardenas, J. & Lipson, M. Graphene electro-optic modulator with 30 GHz bandwidth. *Nat. Photonics* **9,** 511–514 (2015).

14. Tiecke, T. G., Nayak, K. P., Thompson, J. D., Peyronel, T., de Leon, N. P., Vuletić, V. & Lukin, M. D. Efficient fiber-optical interface for nanophotonic devices. *Optica* **2,** 70-75 (2015).
